# Evolution of Hydraulic Conductivity of Unsaturated Compacted Na-Bentonite under Confined Condition—Including the Microstructure Effects

**DOI:** 10.3390/ma15010219

**Published:** 2021-12-28

**Authors:** Tian Chen, Mao Du, Qiangling Yao

**Affiliations:** 1 Key Laboratory of Deep Coal Resource Mining (CUMT), Ministry of Education, Xuzhou 221116, China; tian.chen20@outlook.com (T.C.); yaoqiangling@126.com (Q.Y.); 2 Department of Mechanical, Aerospace and Civil Engineering, School of Engineering, The University of Manchester, Manchester M13 9PL, UK; 3 Department of Earth and Environmental Sciences, School of Natural Sciences, The University of Manchester, Manchester M13 9PL, UK

**Keywords:** unsaturated hydraulic conductivity, compacted Na-bentonite, macro porosity, U-shaped relationship, relative hydraulic conductivity, Kozeny–Carman equation

## Abstract

Compacted bentonite is envisaged as engineering buffer/backfill material in geological disposal for high-level radioactive waste. In particular, Na-bentonite is characterised by lower hydraulic conductivity and higher swelling competence and cation exchange capacity, compared with other clays. A solid understanding of the hydraulic behaviour of compacted bentonite remains challenging because of the microstructure expansion of the pore system over the confined wetting path. This work proposed a novel theoretical method of pore system evolution of compacted bentonite based on its stacked microstructure, including the dynamic transfer from micro to macro porosity. Furthermore, the Kozeny–Carman equation was revised to evaluate the saturated hydraulic conductivity of compacted bentonite, taking into account microstructure effects on key hydraulic parameters such as porosity, specific surface area and tortuosity. The results show that the prediction of the revised Kozeny–Carman model falls within the acceptable range of experimental saturated hydraulic conductivity. A new constitutive relationship of relative hydraulic conductivity was also developed by considering both the pore network evolution and suction. The proposed constitutive relationship well reveals that unsaturated hydraulic conductivity undergoes a decrease controlled by microstructure evolution before an increase dominated by dropping gradient of suction during the wetting path, leading to a U-shaped relationship. The predictive outcomes of the new constitutive relationship show an excellent match with laboratory observation of unsaturated hydraulic conductivity for GMZ and MX80 bentonite over the entire wetting path, while the traditional approach overestimates the hydraulic conductivity without consideration of the microstructure effect.

## 1. Introduction

Bentonite is a widely considered swelling clay as an engineered barrier material in geological waste disposal because it has a large percentage of smectite (50~90%), a clayey soil swelling under water intrusion. Bentonite has lower hydraulic conductivity and diffusivity, and higher expansive capacity, specific surface area, cation-exchange potential, and thermal conductivity compared to other soils [1,2,3,4,5,6]; therefore, it plays a key role in various geo-environmental engineering applications, such as containment systems, carbon dioxide storage [7,8], bioremediation [9], and stability of petroleum reservoirs [10]. Bentonite has been attracting attention in the construction of disposal repository of high-level nuclear waste for 4 decades [11]. Sodium bentonite such as GMZ and MX80 bentonite is commercialised bentonite used more extensively than calcium bentonite in engineering because of its more outstanding expansive ability and lower hydraulic conductivity to water transport [4]. The bentonite could have side effects on personal health because it can prevent digestion and influence the absorption of electrolytes [12]. High levels of germs or heavy metals from bentonite are harmful to health [12]. Meanwhile, the wetting of bentonite clay can damage roadways and buildings without proper pretreatment [13].

In engineering applications, bentonite is normally densely compacted with very low water content, and thus it is unsaturated with a rather high suction at the beginning. Bentonite is placed between the radioactive waste and the host rock, serving as buffer/backfill material. Thereafter, bentonite consistently experiences water intrusion from the host rock. The stiffness and strength of host rock are usually too high to deform, so the wetting path of bentonite happens in a quasiconstant volume condition [14]. Having a solid understanding of hydraulic behaviour of engineering buffer (bentonite) has practical significance in a real disposal repository of HLRW that includes a confined condition and wetting path [14,15,16,17,18,19,20]. Laboratory observations found the water flow and ion diffusion are considerably influenced by the expansion and shrinkage of the microstructure of clays. The application of the conventional constitutive relationship of unsaturated hydraulic conductivity might overestimate the rate of water flow and ion transport [21,22]. Limited experimental and theoretical approaches can provide a solid demonstration of microstructure effects on unsaturated hydraulic conductivity of compacted bentonite. The traditional method for calculating the unsaturated hydraulic conductivity of soils is the product of relative hydraulic conductivity and saturated hydraulic conductivity [23,24,25,26,27,28,29,30,31]; however, both variables are strongly impacted by the swelling microstructure of compacted bentonite [23,32].

Experimental studies show that saturated hydraulic conductivity of porous media is decided by the percentage of smectite, fluid, temperature, porosity, pore pressure and pore geometry that consists of pore size distribution, tortuosity, pore throats, coordination number of pore, etc. [33,34,35,36,37,38]. Despite many efforts to describe saturated hydraulic conductivity [30,39,40,41,42,43], a solid theoretical model of hydraulic properties of swelling clays remains a challenge. A well-developed constitutive model to estimate saturated hydraulic conductivity of coarse-grain and nonswelling soils is the Kozeny–Carman (KC) relationship [44,45]. The KC equation interprets the saturated hydraulic conductivity as a relationship of porosity (*Φ*), specific surface area (*S_A_*), tortuosity (*τ*) and a shape factor (*C_s_*), shown as Equation (1) below,

(1) $$k_{sat}=\frac{C_{s}\gamma_{w}}{\eta \rho_{d}^{2}\tau^{2}S_{A}{}^{2}}\frac{\phi^{3}}{{\left(1-\phi \right)}^{2}}$$

where *k_sat_* is the hydraulic conductivity of saturated soils (m/s), *Φ* is the porosity of soils (dimensionless), *C_s_* is a dimensionless shape constant (dimensionless), *S_A_* is the specific surface area of soil (m^2^/g), *γ_w_* is unit weight of fluid (N/m^3^), *ρ_d_* is the dry density of soil (kg/m^3^), and *η* is fluid viscosity (N∙s/m^2^).

The KC equation was proposed on the hypothesis of fluid through the uniform channels of a cross-section, and the laboratory observation of coarse-grained soils (e.g., sand) was in line with the results calculated by the KC equation [42,45,46]. Since the KC equation assumes no change of soil fabric and no water transport in the solid phase of particles during the wetting path, it is not valid for clayey soils [28,39,47]. The KC equation suggests that saturated hydraulic conductivity (*k_sat_*) versus *Φ*^3^/(1 *− Φ*)^2^ should be a straight line for soils. Nonetheless, experimental results failed to identify such a linear relationship in clayey soils without consideration of the microstructure effects [28].

To obtain the saturated hydraulic conductivity of swelling soils, the microstructure evolution needs to be introduced into the KC equation, mainly including the effective (external) specific surface area, effective (macro) porosity and tortuosity for water flow. Macro pores consist of interparticle and interaggregate pores for compacted clays. Diverse approaches are applied to measure the specific surface such as Grain Size Distribution Curve [47,48], despite the fact that they are not widely employed in soil mechanics and engineering hydraulics. Such approaches work for granular soils without nonswelling fine particles. Although Chapuis and Aubertin [47] presented a new method to estimate specific surface area that fit experimental data very well, it is inappropriate for clays containing smectite. The discrepancy can be ascribed to the swelling capability of clayey soils, resulting in a considerable decline of the external specific surface. In this paper, the stacked lamellar structure of bentonite is further developed based on Holmboe et al. [49] and Tournassat et al. [50], to estimate effective (external) specific surface area for water flow.

The effective (macro) porosity of bentonite is not constant because of the expansion of microstructure after water absorption. Very limited studies have been found in the theoretical description of pore system evolution of wetting bentonite. Likos and Wayllace [51] as pioneers demonstrated a simplified geometrical method to derive the macro porosity, while Sedighi and Thomas [52] proposed a novel approach to describe macro porosity by a geochemical model. To investigate the evolution of macro porosity, the interaction between water and bentonite needs to be clarified. There are three types of water states in compacted bentonite, which incorporates interlayer water located between the stacked Tetrahedral–Octahedral–Tetrahedral layers (TOT layers) of bentonite, water in diffuse double layers and free water [53]. The interlayer water is immobile, while water in the interparticle and interaggregate pores can transport freely. Water in diffuse double layers is partly restrained with much larger mobility than the interlayer water. During the wetting path, bentonite will absorb water, most of which enters interlayer pores of particles, leading to the decease of macro porosity. To predict the saturated hydraulic conductivity of compacted clays, the macro porosity needs to be applied instead of total porosity according to the realistic description of water states in the pore system of compacted clays. 

Two well-known approaches employed to derive the relative hydraulic conductivity were proposed by Mualem [54] and Brooks and Corey [55] in this field. Mualem [54] described relative hydraulic conductivity as a relationship of volumetric water content as shown in Equation (2), while Brooks and Corey [55] suggested relative hydraulic conductivity as a function of suction (Equations (3)–(5)). Both of the traditional models ignore the microstructural expansion of compacted bentonite, leading to an overestimation of hydraulic conductivity and failure in explanation of the decrease of hydraulic conductivity in the early stage of wetting path (Ye et al 2009; Cui et al, 2008; Chen et al, 2020). Subsequently, van Genuchten [24] proposed a renowned water retention function that makes suction and volumetric water content convertible. In experimental observations, the hydraulic conductivity of compacted bentonite against suction shows a U-shaped curve that cannot be captured by the two traditional methods with a monotonic function; therefore, the microstructure effect needs to be taken into account to explain the observation,

(2) $$k_{r}=S_{e}^{\alpha }=(\frac{\theta -\theta_{r}}{\theta_{sat}-\theta_{r}}{)}^{\alpha }$$

where *k_r_* is relative hydraulic conductivity, *k_sat_* is the saturated hydraulic conductivity, *k_unsat_* is the unsaturated hydraulic conductivity, *S_e_* is the effective saturation, *θ* and *θ_r_* are the actual and the residual volumetric water content, respectively, and α is a constant parameter, which is assumed to be 3.5 as an average widely adopted for soils in the literature [55,56],

(3) $$k_{r}=\frac{k_{unsat}}{k_{sat}}=S_{e}^{2}\frac{\int_{0}^{S_{e}}\psi^{-(1+\frac{1}{\mu })}dS_{e}}{\int_{0}^{1}\psi_{}^{-(1+\frac{1}{\mu })}dS_{e}}$$

(4) $$S_{e}={\left(\frac{\psi }{\psi_{d}}\right)}^{-\lambda }for\;\psi <\psi_{d}$$

(5) $$S_{e}=1for\;\psi \geq \psi_{d}$$

where *μ* is a dimensionless number, *Ψ* is suction and *Ψ_d_* is the air entry value, and *λ* is a fitting factor related to pore-size distribution.

In this paper, compacted bentonite is applied to explore the microstructure evolution of expansive clays. This work aims to address the challenge in the overestimation of hydraulic properties of bentonite because of the pore network evolution and explore the theoretical method to describe the U-shaped hydraulic conductivity by considering both the microstructure evolution and dropping gradient of suction. The new theoretical model is proposed to describe the key hydraulic parameters with the consideration of microstructure effects, including the evolution of the specific surface area, porosity and tortuosity over wetting path. The investigation of pore evolution starts with the clay–water–chemical interaction in the stacked structure of compacted bentonite. The dynamic process of water absorption is discussed to estimate the amount of water in interlayer pores and macro pores and pore network evolution over the hydration process. The revised KC equation is proposed for predicting the saturated hydraulic conductivity of compacted bentonite by introducing the modified hydraulic parameters. The relative hydraulic conductivity is further developed to form a constitutive relationship that consists of the influence of suction and microstructure effects, followed by the comparison with experimental results.

## 2. Theory and Methodology

### 2.1. Pore Evolution of Compacted Bentonite

#### 2.1.1. Specific Surface Area

**Figure 1:**
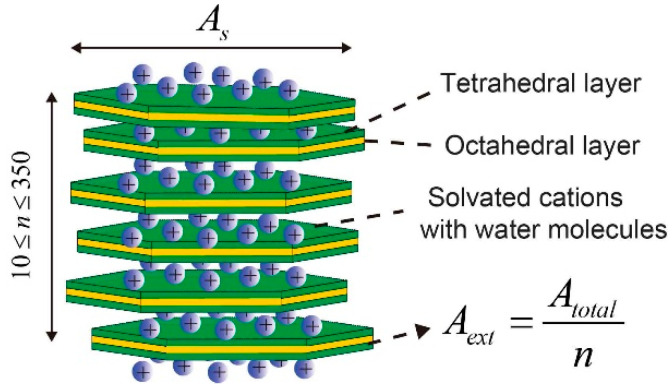
A schematic diagram of lamellar structures of sodium bentonite.

Figure 1 shows a typical lamellar structure of one bentonite particle that consists of 10 to 350 TOT layers (Tetrahedral–Octahedral–Tetrahedral layers). The TOT layers of bentonite are stacked because of repulsive and attractive forces, forming crystal particles. The external surface area (*A_ext_*) contributes to overall flux instead of total surface area (*A_tot_*). *A_tot_* that is independent of water content can be measured by experimental techniques such as Ethylene Glycol Monoethyl Ether. The relationship between *A_ext_* and *A_tot_* was given as Equation (6) according to the lamellar structure of smectite [50,57,58,59,60],

(6) $$A_{ext}=\frac{A_{tot}}{n}$$

where *n* is the stacked TOT layers of 1 particle with a range from 10 to 350 depending on water content and the type of bentonite sample.

To estimate the evolution of pore structure, the surface area (*A_s_*) of a single TOT layer in particles (*A_s_*) needs to be assessed. The external specific surface area is defined as the external surface of a particle divided by the mass of the particle,

(7) $$A_{\mathrm{ext}}=\frac{2A_{s}}{m_{\mathrm{single}}}$$

where *m_single_* is the average mass of per particle. 

The total number of TOT layers (*N*) in a bentonite sample is described as Equation (8).

(8) $$N=\frac{m_{\mathrm{smectite}}}{m_{\mathrm{single}}}n$$

where $m_{\mathrm{smectite}}$ is the mass of smectite (kg).

Therefore, the *m_single_* is expressed as below,

(9) $$m_{\mathrm{single}}=\frac{m_{smectite}n}{N}$$

Substituting Equation (9) to Equation (7) yields,

(10) $$A_{ext}=\frac{2A_{s}}{m_{smectite}n/N}$$

Therefore, the combination of Equations (6) and (10) yields *A_s_*,

(11) $$A_{s}=\frac{m_{smectite}A_{tot}}{2N}$$

#### 2.1.2. Evolution of Pore System

**Figure 2:**
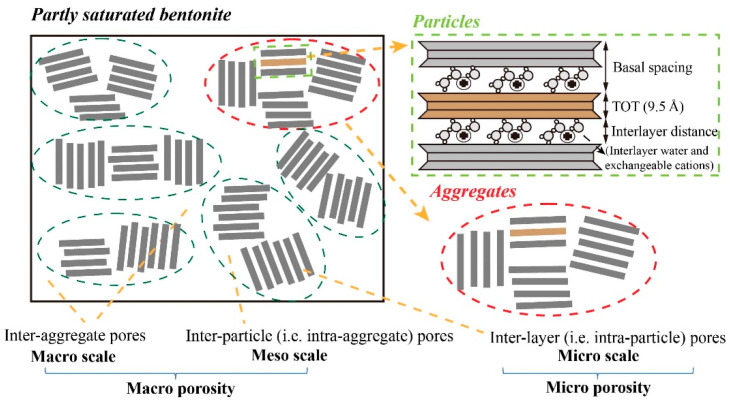
Conceptual diagram of pore system of compacted bentonite.

Figure 2 presents a conceptual diagram of the pore system of compacted bentonite. The microstructure of compacted bentonite is composed of a cluster of TOT layers that constitute bentonite particles and aggregates [52,61]. In many natural porous materials, such as tight rocks or compacted swelling clays, the intraparticle and interparticle pore sizes are in the range of micro (less than 5 nm), meso (between 5 and 50 nm), and macro pores (more than 50 nm) [62]. Several different pore definitions for compacted bentonite were displayed in different publications [19,63,64] where the interlayer pores are identical to intraparticle pores and interparticle pores referred as to intra-aggregate pores. Water flow is constrained in interlayer pores because of interaction between water and the TOT surface of compacted bentonite; therefore it reaches a consensus that the water in interlayer pores is immobile [53,65]. By contrast, water can freely flow through interparticle and interaggregate pores, which contribute to long-range water transport. The interparticle and interaggregate pores are collectively referred to as macro pores, as shown in Figure 2.

In laboratory observations, the number of stacked water layers between two adjacent TOT layers was summarised as one, two, three and even four with higher water content, as shown in Table 1 and Table 2, in which the basal spacing is the summation of the thickness of one TOT layer and thickness of water layers between TOT layers (see the upper-right panel of Figure 2). The four water layers have not been extensively measured and accepted until now, which was found by Saiyouri et al. [66] using MX-80 bentonite. Table 1 shows that the thickness of a single TOT layer is mostly reported as 9.5 Å and the single water layer thickness is 3 Å. The diameter of a water molecule is reported as 3 Å at standard temperature and pressure [67,68], which is identical to the thickness of a single water layer presented in Table 1. Accordingly, the maximal basal spacing is the summation of three water layers and one TOT layer, equal to 18.5 Å, which is consistent with the summary of Table 1.

The adsorption reaction rate of interlayer water is comparable with hydrodynamic adsorption, which is presented by a pseudo-first order kinetic model [73]. Therefore, the evolution of basal spacing against water content is developed based on the hydrodynamic adsorption equation,

(12) $$\frac{dq_{t}}{dt}=k_{a}(q_{e}-q_{t})$$

where *q_e_* and *q_t_* (kg/kg) are the mass of absorbed solute at equilibrium and at time *t* (h), and *k_a_* (h^−1^) is the rate constant of the pseudo-first order kinetic adsorption. As stated by the boundary conditions (*t* = 0, *q_t_* = 0; *t* = *t*, and *q_t_* = *q_t_*), the integral of the hydrodynamic adsorption equation yields,

(13) $$q_{t}=q_{e}(1-e^{-k_{a}t})$$

If the water layers are stacked in order then,

(14) $$\left(d_{t}-d_{TOT})=(d_{e}-d_{TOT})(1-e^{k_{a}t}\right)$$

**Figure 3:**
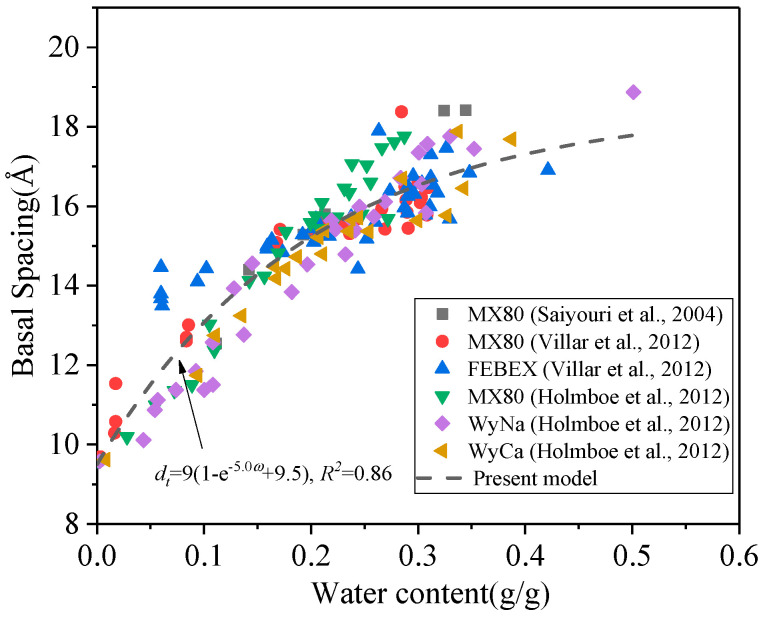
Relationship between basal spacing and water content.

If the water content (*ω*, kg/kg) is assumed to have a linear relationship with time, then yields,

(15) $$d_{t}=(d_{e}-d_{TOT})(1-e^{bt})+d_{TOT}$$

(16) $$d_{t}=9(1-e^{-5.0\omega })+9.5,R^{2}=0.860$$

where *b* is a constant correlating with water absorption rate, which is −5.0 obtained by the experimental data (see Figure 3), *d_TOT_* and *d_t_* are the thickness of a single TOT layer and the basal spacing, respectively, *e* is natural logarithm.

The water content can be converted into volumetric water content,

(17) $$\theta =\frac{V_{w}}{V_{t}}=\frac{m_{w}}{\rho_{w}}\frac{1}{V_{t}}=\frac{m_{s}\omega }{\rho_{w}}\frac{1}{\frac{m_{s}}{\rho_{d}}}=\frac{\rho_{d}}{\rho_{w}}\omega$$

where *m_s_* is the mass of solid (kg), *m_w_* is the mass of water (kg), *ω* is the water content (kg/kg), *ρ_w_* is the density of water (kg/m^3^), *V_t_* is the total volume of the sample, *V_w_* is the volume of total water in the sample (m^3^). 

In a bentonite particle, the thickness of interlayer water between two TOT layers ($d_{iw}$) can be calculated as follows,

(18) $$d_{iw}=d_{t}-d_{TOT}$$

The volume of immobile water (namely, the volume of interlayer water, *V_imw_*) can be estimated,

(19) $$V_{imw}=A_{s}d_{iw}N=\frac{1}{2}d_{iw}A_{tot}m_{s}^{s}$$

Consequently, the volume of macro pores (*V_macro_*) is derived,

(20) $$V_{macro}=V_{t}-V_{imw}-V_{s}$$

where *V_s_* is the volume of solid.

Therefore, the macro porosity (*Φ_macro_*) can be calculated,

(21) $$\phi_{\mathrm{macro}}=\frac{V_{e}}{V_{t}}=1-\frac{V_{s}}{V_{t}}-\frac{d_{iw}A_{tot}m_{s}^{s}}{2V_{t}}$$

Accordingly,

(22) $$\phi_{\mathrm{total}}=1-\frac{\rho_{d}}{G_{s}}$$

(23) $$\phi_{\mathrm{micro}}=\frac{d_{iw}A_{tot}\rho_{d}^{s}}{2}$$

(24) $$\phi_{\mathrm{macro}}=1-\frac{\rho_{d}}{G_{s}}-\frac{d_{iw}A_{tot}\rho_{d}^{s}}{2}$$

where *Φ_macro_* is the macro porosity (i.e., effective porosity), *Φ_micro_* is the micro porosity (i.e., porosity of interlayer water), $\rho_{d}^{s}$ is the dry density of the smectite (kg/m^3^), *G_s_* is the specific gravity of soil, *ρ_d_* is the dry density of the soil (kg/m^3^).

Bentonite contains impurities (i.e., non-smectite portions), for instance, the GMZ bentonite consisting of 75.4% smectite and 24.6% impurities (11.7% quartz, 7.3% Cristobalite, 4.3% feldspar, 0.8% Kaolinite, 0.5% calcite, etc.) [17]. The dry density of smectite can be derived using Equation (25), if the mass fraction of smectite in the bentonite (*X_sm_*, kg/kg) can be obtained [74],

(25) $$\rho_{d}^{s}=X_{sm}\rho_{d}[1-(1-X_{sm})\frac{\rho_{d}}{\rho_{im}}){]}^{-1}$$

where *ρ_im_* denotes the density of the non-smectite minerals or impurities (kg/m^3^).

In summary, the macro porosity only correlates with one unknown variable, i.e., volumetric water content,

(26) $$\phi_{\mathrm{macro}}=1-\frac{\rho_{d}}{G_{s}}-\frac{9}{2}A_{tot}X_{sm}\rho_{d}[1-(1-X_{sm})\frac{\rho_{d}}{\rho_{im}}){]}^{-1}(1-e^{-5.0\frac{\rho_{w}}{\rho_{d}}\theta })$$

where *ρ_i_* is a constant that was recommended 2.8 kg/m^3^ by literature [52,74].

#### 2.1.3. Tortuosity

Numerous studies have indicated that pore tortuosity serves as a non-negligible factor in determining the hydraulic conductivity of soil [6,35,45,75,76,77,78,79,80]. Pore tortuosity is defined as the ratio of the effective path length (*L_e_*) to the sample length (*L*) [45,81,82],

(27) $$\tau =L_{e}/L$$

Carman [45] proposed the first derivation of tortuosity as $L_{e}/L=sec\left(\alpha \right)$, where α is the angle between the apparent direction of flow and flow pathway for stacked spheres, α is assumed as 45°, thus $L_{e}/L$ equals $\sqrt{2}$. Tortuosity of porous media has a logarithmic relationship with porosity, reported as $\tau^{2}=1-pln(\phi ),$ where *p* is a fitting constant [78,83]. Regarding swelling soils with fine granules and lamellar structures, $\tau^{2}=1-1/2ln(\phi )$ given by Weissberg [84] is in line with experimental data [83,84,85].

### 2.2. Model for Unsaturated Hydraulic Conductivity

In the hypothesis of this work, the saturated hydraulic conductivity of compacted bentonite (*k_sat_*) can be derived by taking into account microstructure effects,

(28) $$k_{\mathrm{sat}}=\frac{C_{s}\gamma }{\eta \rho_{d}^{2}\tau^{2}A_{ext}{}^{2}}\frac{\phi_{macro}^{3}}{{\left(1-\phi_{\mathrm{macro}}\right)}^{2}}$$

where *γ* is the unit weight of the fluid (N/m^3^) and *η* is the fluid viscosity (N s/m^2^) [39,86]. Shape factor (*C_s_*) is reported as 0.2 for soils [33,42,45,87,88].

The unsaturated hydraulic conductivity (*k_unsat_*) is the product of relative hydraulic conductivity (*k_r_*) and saturated hydraulic conductivity (*k_sat_*) [55],

(29) $$k_{unsat}=k_{r}k_{sat}$$

The combination of Equations (2)–(5) yields the relative hydraulic conductivity as follows,

(30) $$k_{r}={\left(\frac{\psi }{\psi_{d}}\right)}^{-(1/\mu +1+3\lambda )}$$

and,

(31) $$k_{r}=S_{e}{}^{3+\frac{1+1/\mu }{\lambda }}$$

The pore geometry of nonswelling soils is considered a constant whose unsaturated hydraulic conductivity is only impacted by the change of suction. Nevertheless, in reality, the unsaturated hydraulic conductivity of bentonite will be considerably affected by changing hydraulic parameters, such as macro porosity and external specific surface area and tortuosity, caused by the microstructure evolution of the pore network. In early studies, there was a lack of a cutting-edge laboratory device to measure the unsaturated hydraulic conductivity of low-permeability clays such as bentonite. The recent observations showed unsaturated hydraulic conductivity against suction should be a U-shaped curve instead of a monotonically decreasing function [15,89,90]. Therefore, this work considers swelling effects, differing from the traditional constitutive relationship such as Equations (30) and (31) where only suction influences the hydraulic conductivity. If there are swelling effects, a reference point where hydraulic conductivity is equal to fully saturated hydraulic conductivity can be found [23],

(32) $$k_{r}^{*}=\frac{k_{\mathrm{unsat}}}{k_{sat}}=\frac{k_{\mathrm{unsat}}}{k_{unsat,ref}}=\frac{k_{r}}{k_{r,ref}}\frac{k_{\mathrm{sat}}}{k_{sat,ref}}$$

The combination of Equations (31) and (32) yields,

(33) $$k_{r}^{*}={\left(\frac{\psi \psi_{d,ref}}{\psi_{d}\psi_{ref}}\right)}^{-(1/\mu +1+3\lambda )}\left(\frac{k_{sat}}{k_{sat,ref}}\right)$$

The subscript *ref* denotes the reference point where *k_unsat,ref_* equals *k_sat_* (namely *k_r_* = 1) in the U-shaped curve observed in swelling clays. *Ψ_ref_* is the suction at the reference point. All the corresponding *k_unsat,ref_*, *Ψ_ref_* and *Φ_ref_* are the values at the same reference point. One example can be found for Na-bentonite Kunigel-V1 in Cui et al. [15], where the suction is 35 MPa and corresponding *k_r_* = 1 as the reference point used by Liu et al. [23].

The expansive capability results in the decrease of macro porosity. Leverett [91] proposed that the change of air entry value (*Ψ_d_*) can be represented by a function of porosity under confined condition. Therefore, Liu et al. [23] deduced Equations (34) and (35) to establish the relationship of *Ψ_d_* and air entry value at the reference point (*Ψ_d,ref_*) under the confined condition as below,

(34) $$\frac{\psi_{d,\mathrm{ref}}}{\psi_{d}}=\frac{\phi }{\phi_{ref}}$$

(35) $$\frac{k_{\mathrm{sat}}}{k_{sat,\mathrm{ref}}}={\left(\frac{\phi }{\phi_{ref}}\right)}^{(1+1/\mu )\sigma }$$

where parameter *σ* is larger than one for swelling materials because the porosity may not reflect the effect of well-connected pores on hydraulic conductivity.

Substituting Equations (34) and (35) into Equation (33) yields,

(36) $$k_{r}{}^{*}={\left(\frac{\psi }{\psi_{ref}}\right)}^{-(1/\mu +1+3\lambda )}{\left(\frac{\phi }{\phi_{ref}}\right)}^{(\sigma -1)(1+1/\mu )-3\lambda }$$

Comparing Equation (36) with Equations (34) and (35), the revised model introduces one more factor related to porosity, i.e., ${\left(\phi /\phi_{ref}\right)}^{\left(\sigma -1\right)\left(1+1/\mu \right)-3\lambda }$ which represents the expansive capability of clayey soil under constant-volume condition, and ${\left(\psi /\psi_{ref,c}\right)}^{-\left(1/\mu +1+3\lambda \right)}$ reflects the change of hydraulic conductivity resulting from declining suction.

Pham et al. [92] reported that the swelling capability of clays was an exponential function of suction under confined condition,

(37) $$\frac{\phi }{\phi_{ref}}=\exp\left(\beta (\frac{\psi }{\psi_{ref}}-1)\right)$$

where *β* is a fitting factor.

The combination of Equations (36) and (37) yields the constitutive relationship of unsaturated swelling clays as below,

(38) $$k_{r}{}^{*}={\left(\frac{\psi }{\psi_{ref}}\right)}^{-(1/\mu +1+3\lambda )}\exp{\left(\left(\frac{\psi }{\psi_{ref}}-1\right)\right)}^{\beta [(\sigma -1)(1+1/\mu )-3\lambda ]}$$

where the experimental data reported in Cui et al. [15] support that *μ* equals 0.28 as a constant with various suction; *β* can be derived by Equation (37), while The σ and *λ* can be found using experimental data.

## 3. Results and Discussion

### 3.1. Experimental Data and Parameters of Model

In the work, the experimental materials are employed to verify the theoretical model including Na-bentonite GMZ and MX80 whose properties are listed in Table 3.

**Figure 4:**
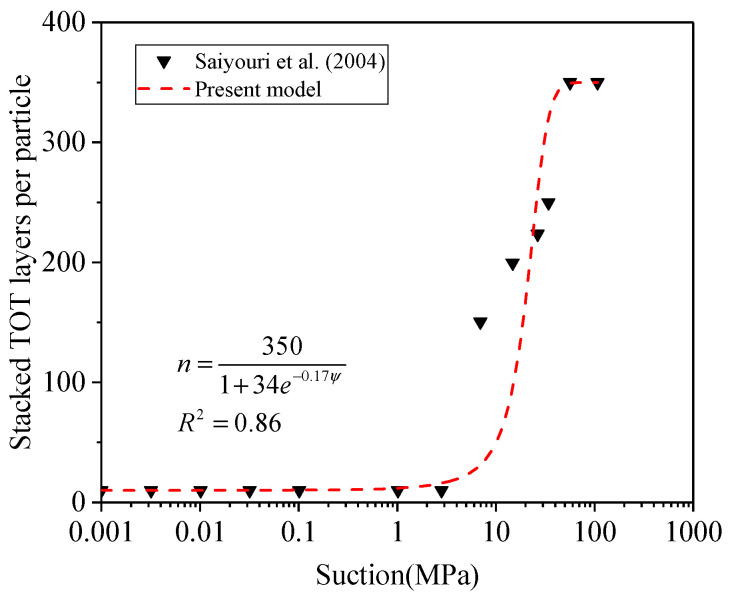
Relationship between stacked TOT layers per bentonite particle and suction.

External specific surface area is the first parameter estimated to derive the saturated hydraulic conductivity based on the revised Kozeny–Carman equation. The experimental data of stacked TOT layers per particle were measured by Saiyouri et al. [66]. A fit to the data was proposed by Chen et al (2020), showing a good match,

(39) $$n=\frac{n_{\max}}{\left(1+(n_{\max}/n_{\min}-1)e^{c\psi }\right)}$$

where *n*_max_ and *n*_min_ are the maximum and minimum stacked TOT layers per particle with 10 and 350, respectively, given by Saiyouri et al. [66], and *c* is a dimensionless fitting parameter that is equal to −0.17 for Na-bentonite according to Figure 4 and is associated with the reaction rate between water and bentonite.

Consequently, the external specific area can be illustrated,

(40) $$A_{ext}=\frac{A_{tot}}{n_{c}}=\frac{570}{350/(1+34e^{-0.17\psi })}$$

**Figure 5:**
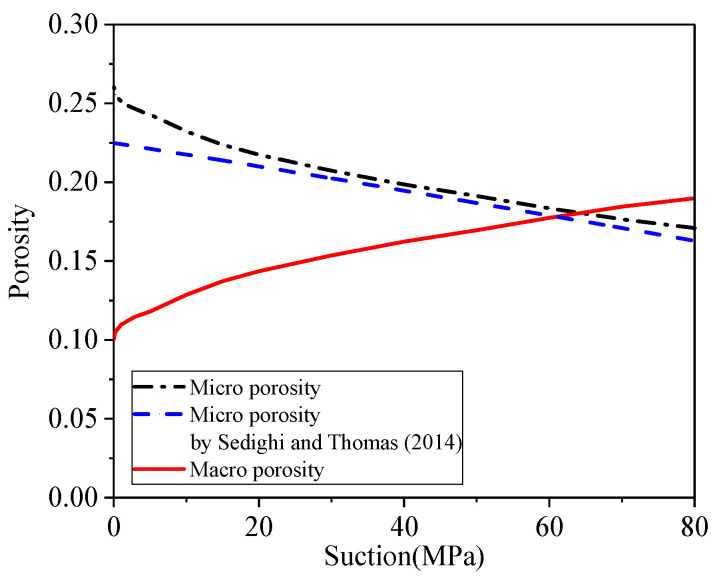
Macro and micro porosity against suction for compacted bentonite.

To derive macro porosity (effective porosity), the data from Table 3 are substituted into Equation (26), and the results are shown in Figure 5 (the red line). To evaluate its accuracy, this work compares with the theoretical method of micro porosity proposed by Sedighi and Thomas [52] as Equation (41),

(41) $$\phi_{micro}=X_{hs}\frac{\zeta_{c}\upsilon_{il}}{M_{sm}}\rho_{d}^{s}$$

where *X_hs_* denotes the mole fraction of hydrated smectite, which can be gained from Sedighi and Thomas [52]; *M_sm_* is the molar mass of dry smectite (kg/mol), given as 378.787 kg/mol by Gailhanou et al. [94]; *ζ_c_* is the number of moles of water in the interlayer adsorption or desorption reaction, reported as 4.5 mol of water, if a maximum of two monolayers of adsorbed water occurs in the interlayer pores [52]; and *υ_il_* is the molar volume of the interlayer water, recommended as 17.22 m^3^/mol [95].

The outcomes from the two presented methods are compatible for compacted MX-80 bentonite. However, the proposed method has an advantage in feasibility compared with the geochemical model proposed by Sedighi and Thomas [52] because of fewer assumptions during the process of derivation. Moreover, in comparison with two water layers between TOT layers in Sedighi and Thomas [52], this present method takes into account three water layers that are closer to the recent experimental observation for Na-bentonite. 

Figure 5 shows the *Φ_micro_* increases with a decrease in suction and finally approaches stable at 0.260, while the *Φ_macro_* drops before stable at 0.101. In the fully saturated condition, *Φ_micro_* is 2.6 times higher than *Φ_macro_* that only makes up 27.9% of the total porosity (0.361). As a summary, as fewer than 30% of pores are macro pores that contribute to long-term water flow, the traditional KC equation using total porosity will largely overestimate the saturated hydraulic conductivity of bentonite. Secondly, the difference between macro porosity and total porosity explains clayey soils such as bentonite with a large porosity but much lower permeability compacted with nonexpansive soils. Thirdly, the transfer from *Φ_macro_* to *Φ_micro_* over a large range of suction gives rise to a decrease of unsaturated hydraulic conductivity, and as stated above, *Φ_micro_* makes no contribution to long-range water transport.

### 3.2. Saturated Hydraulic Conductivity

Since only macro pores contribute to water flow in compacted bentonite, the total porosity in the Kozeny–Carman equation is replaced by the macro parameter that includes external specific surface area, macro porosity and tortuosity. These macro parameters change with the swelling microstructure of compacted bentonite in the wetting path:

(42) $$k_{\mathrm{sat}}=\frac{C_{s}\gamma }{\eta \rho_{m}^{2}\tau_{\mathrm{macro}}{}^{2}A_{ext}^{2}}\frac{\phi_{\mathrm{macro}}^{3}}{{\left(1-\phi_{\mathrm{macro}}\right)}^{2}}$$

The variables or constants from Table 3 are substituted to Equation (42), which yields that *k_sat_* of GMZ equals 1.01 × 10^−13^ (m/s). Based on the macro porosity derived from Sedighi and Thomas [52], the *k_sat_* of GMZ is 1.88 × 10^−13^ (m/s), whereas the experimental result of *k_sat_* is 1.18 × 10^−13^ [14]. The calculated results fall within the range from 1/3 to 3 times of experimental values, which are regarded as acceptable outcomes because of the uncertainty of experimental hydraulic conductivity [28,33,86,87,96]. In summary, both methods achieve reliable results of estimated saturated hydraulic conductivity that fall within the acceptable experimental range, whereas the predictive results of the present method are closer to experimentally measured values.

### 3.3. Unsaturated Hydraulic Conductivity

**Figure 6:**
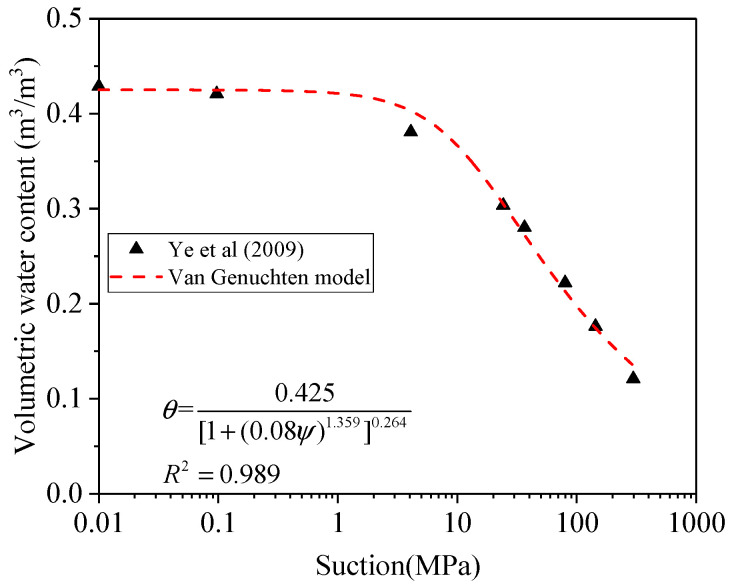
Soil water retention curve.

In this section, the relative hydraulic conductivity ($k_{r}{}^{*}$) of Na-bentonite including GMZ bentonite and MX80 bentonite is discussed in order to predict the unsaturated hydraulic conductivity. The reference point where *k_unsat_* = *k_sat_* has not been found for GMZ bentonite by experiments (Ye et al., 2009); however, it can be estimated that when suction is approaching 100 MPa (*Ψ_ref_*), *k_unsat_* equals *k_sat_*, based on the trend of experimental hydraulic conductivity. Since the suction can be presented by volumetric water content according to van Genuchten [24], the macro porosity is derived using the volumetric water content through Equation (26). As van Genuchten [24] reported, the *θ_r_* of clayey soil can be regarded as zero, and the *θ_s_* of GMZ is 0.425 (m^3^/m^3^) derived from experimental data of Ye et al. [14]. The fitting parameters “*a*” and “*m*” can be acquired from Figure 6:

(43) $$\theta =\frac{\theta_{s}-\theta_{r}}{{\left[1+{\left(a\psi \right)}^{1/(1-m)}\right]}^{m}}+\theta_{r}$$

(44) $$\theta =\frac{0.425}{{\left[1+{\left(0.08\psi \right)}^{1.359}\right]}^{0.264}}$$

**Figure 7:**
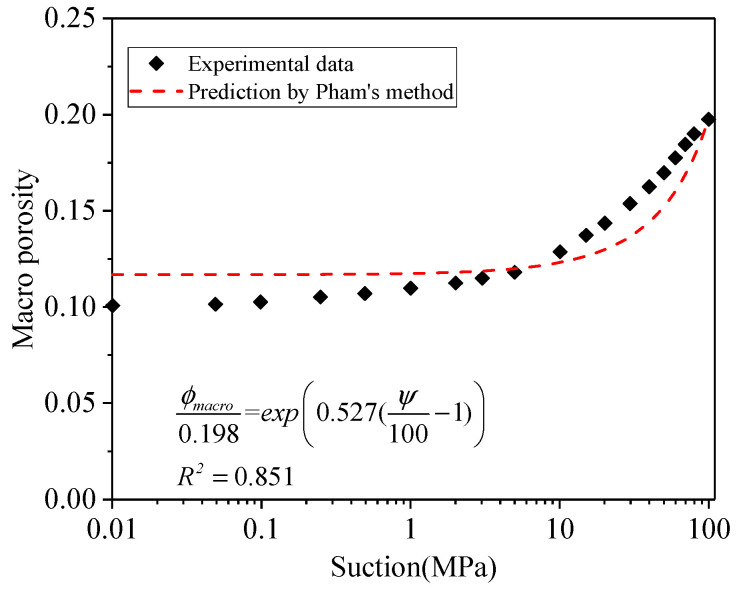
Relationship between macro porosity and suction.

Consequently, when suction is 100 MPa (*Ψ_ref_*), the *θ_ref_* is corresponding to 0.209. The combination of Equations (37) and (44) yields *Φ_ref_* as 0.198. Therefore,

(45) $$\frac{\phi_{\mathrm{macro}}}{0.198}=\exp\left(\beta (\frac{\psi }{100}-1)\right)$$

where *β* is estimated as 0.537 from Figure 7.

Since the *Ψ_ref_* (100 MPa) is obtained by extending the trend of the experimental curve, it could be slightly different from the real value of *Ψ_ref_*; therefore, a small correction term (*C* × *Ψ/Ψ_ref_*) is introduced into Equation (38), yielding Equation (46), which reduces the deviation caused by the assumed *Ψ_ref_*. If *Ψ_ref_* can be measured accurately, the small correction term can be considered as zero, which has been proved by Liu et al. [23]. In summary, the *C* can be understood as a factor to express the relative error between the estimated *Ψ_ref_* and the actual *Ψ_ref:_*

(46) $$k_{r}{}^{*}={\left(\frac{\psi }{\psi_{\mathrm{ref}}}\right)}^{-(1/\mu +1+3\lambda )}\exp{\left(\left(\frac{\psi }{\psi_{ref}}-1\right)\right)}^{\beta [(\sigma -1)(1+1/\mu )-3\lambda ]}+C\frac{\psi }{\psi_{ref}}$$

(47) $$k_{unsat}=k_{sat}k_{{}_{r}}^{*}=k_{sat}[{\left(\frac{\psi }{\psi_{ref}}\right)}^{-(1/\mu +1+3\lambda )}\exp{\left(\left(\frac{\psi }{\psi_{ref}}-1\right)\right)}^{\beta [(\sigma -1)(1+1/\mu )-3\lambda ]}+C\frac{\psi }{\psi_{ref}}]$$

**Figure 8:**
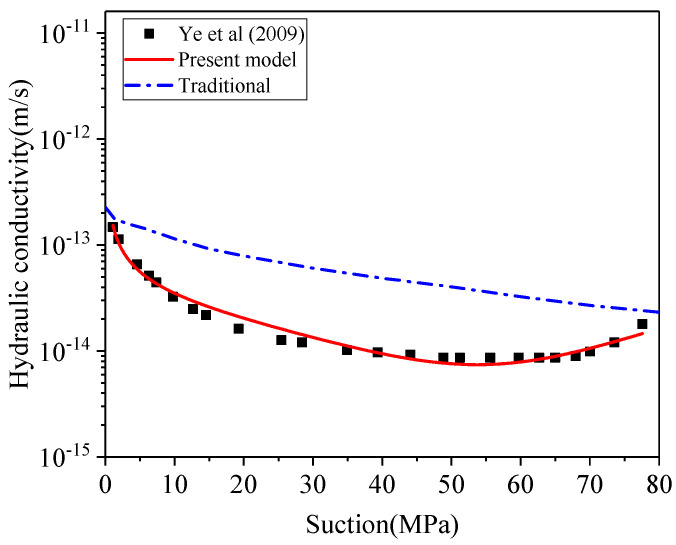
The prediction of unsaturated hydraulic conductivity for GMZ bentonite.

When the experimental data from Ye et al. [14] are substituted into the constitutive model, the *λ*, *σ* and *C* are equal to −1.29, 1.318, −0.64 as shown in Equation (48). From the predicted curve of Figure 8, the degree of correlation between experimental data and prediction is 99.0%, which shows the high accuracy of the theoretical model on *k_unsat_*.

(48) $$k_{unsat}=1.1\times 10^{-13}[{\left(\frac{\psi }{100}\right)}^{-0.70}\exp\left(2.86(\frac{\psi }{100}-1)\right)-0.64\frac{\psi }{100}]$$

**Figure 9:**
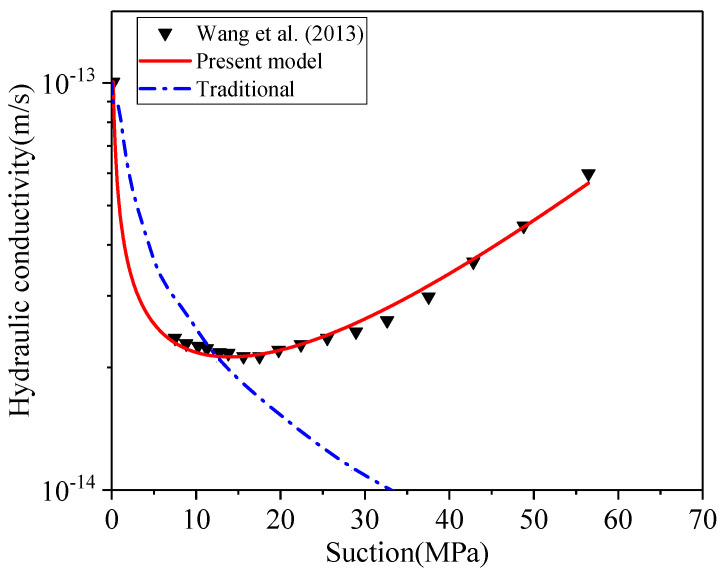
The prediction of unsaturated hydraulic conductivity for MX80 bentonite.

This model is also verified to predict *k_unsat_* using the MX80 bentonite measured by Wang et al. [90]. As shown in Figure 9, the results confirm that the present model can precisely predict the *k_unsat_* of MX80 with a degree of correlation as 99.3%.

(49) $$k_{unsat}=1\times 10^{-13}[{\left(\frac{\psi }{70}\right)}^{-0.46}\exp\left(2.77(\frac{\psi }{70}-1)\right)-0.1\frac{\psi }{70}]$$

The proposed constitutive relationship incorporates the effect of microstructural expansion on the evolution of unsaturated hydraulic conductivity. The outcome of the proposed model is in line with the experimental measurement for both bentonites. The traditional approach (Equations (30) and (31)), which ignores the microstructure effects, yields much higher prediction and is not able to explain the decrease of hydraulic conductivity with declining suction. This developed constitutive relationship can well describe water flow in swelling bentonite that has the U-shaped hydraulic conductivity. This proposed relationship reveals that macro pores (*Φ_macro_*) continue to convert into micro pores (*Φ_micro_*), and the average pore size of bentonite decreases over wetting path (dropping suction) of compacted bentonite, resulting in a drop of hydraulic conductivity. Subsequently, the hydraulic conductivity is dominated by the gradient of suction, showing an increasing trend in the low suction range. As a summary, the predictive outcomes of the new constitutive relationship show an excellent match with laboratory observation of unsaturated hydraulic conductivity, while the traditional approach overestimates the hydraulic conductivity without consideration of microstructure effect.

## 4. Summary and Conclusions

This work aims to develop a new constitutive relationship for unsaturated hydraulic conductivity of compacted bentonite, including the microstructure effect. The microstructure effect is represented by the change of accessible porosity (i.e., macro porosity) for water, further resulting in a varying tortuosity, specific surface area and relative hydraulic conductivity. The modified Kozeny–Carman equation was developed for saturated hydraulic conductivity incorporating the microstructure evolution, yielding a good agreement with experimental results. Furthermore, a new constitutive relationship for relative hydraulic conductivity was developed, including both effects of capillary pressure and microstructural expansions during wetting path. The prediction of the new constitutive relationship is in excellent accord with the laboratory observation for GMZ and MX80 bentonite over the entire wetting path, explaining the initial decrease of hydraulic conductivity with declining suction (i.e., U-shaped curve) for compacted bentonite. The traditional model ignores microstructure expansion, leading to an overestimation of hydraulic conductivity and failure in explanation of decrease of hydraulic conductivity in the early stage of wetting path. This work helps improve the understanding of accurate prediction of flow process in the environmental application of swelling clays, including containment systems, especially in the geological disposal of high-level nuclear waste.

## Figures and Tables

**Table 1:** The number of water layers between two adjacent TOT layers and basal spacing of compacted bentonite.

Water Layers	Basal Spacing (Å)					
	[69]	[70]		[49]		[66]
0	9.5	10	9.5	9.5	9.2–10.1	10
1	12.4	12.5	12.3	12.4	12.2–12.7	12.6
2	15.4	15.5	15.0	15.6	15.2–15.7	15.6
3	18.4	18.5	18.5	18.9	18.4–19	18.6
4	21.6 *	\	\	21.8 *	21.4–22 *	21.6

The asterisk (*) represents the data that were estimated by researchers rather than by their experimental observations.

**Table 2:** The number of water layers with increasing water content for bentonite.

Water Layers	Water Content (g/g, %)				
	[71,72]	[70]	[66]	[49]	
0	<7	<8.6	<11.1	<10.8	<8.8
1	7–20	8.6–16.8	11.1–19.2	10.8–23.3	8.8–19.7
2	10–20	16.8–28.4	19.2–32.4	23.3–35.4	19.7–30.3
3	20–35	>28.4	32.4–69.4	>35.4	>30.3
4	\	\	>69.4	\	\

**Table 3:** The basic properties of GMZ and MX80 bentonite.

Mineral	GMZ	MX80
Montmorillonite (%)	75.4 ^1^	79 ^1^
Particle < 2 μm (%)	60 ^1^	60 ^1^
Specific surface area (m^2^/g)	570 ^1^	756 ^4^
Gs	2.66 ^1^	2.82 ^2^
CEC (meq/100 g)	77.3 ^1^	82.3 ^1^
WL (%)	313 ^1^	519 ^1^
WP (%)	38 ^1^	35 ^1^
IP	275 ^1^	484 ^1^
Molar mass (g/mol O_10_(OH)_2_))	\	378.79 ^3^
1—[15,17]. 2—[93]. 3—[94]. 4—[49].		

## Data Availability

All data, models, or code that support the findings of this study are available from the corresponding author.

## Abbreviations

*k*	Hydraulic permeability (m/s)
*k_r_*	Relative hydraulic conductivity
$k_{r}{}^{*}$	Proposed relative hydraulic conductivity of compacted bentonite
*k_sat_*	The saturated hydraulic conductivity (m/s)
*k_unsat_*	The unsaturated hydraulic conductivity (m/s)
*S_e_*	The effective saturation
*θ*	The actual volumetric water content
*θ_r_*	The residual volumetric water content
*θ_s_*	The saturated volumetric water content
*α*	The constant parameter in Mualem [54]
*μ*	A dimensionless number in Burdine [31]
*Ψ*	Suction of soil/bentonite(MPa)
*Ψ_d_*	The air entry value (MPa)
*λ*	A fitting factor related to pore-size distribution
*C_s_*	Shape factor
*τ*	Tortuosity
*γ_w_*	Unit weight of water (N/m^3^)
*η*	Viscosity of water (Pa∙s)
*S_A_*	Specific surface area (m^2^/kg)
*Φ*	Porosity
*ρ_d_*	Dry density of soil (kg/m^3^)
*A_tot_*	Total specific surface area (m^2^/kg)
*n*	The number of TOT layers of per particle
*N*	Total number of TOT layers of bentonite sample
*A_ext_*	External specific surface area of bentonite (m^2^/g)
*A_s_*	The surface area of a single TOT layer
*m_si_*	Mass of one particle (kg)
*m_smectite_*	Mass of smectite (kg)
*m_s_*	Mass of solid (kg)
*b* *C*	A constant related to water adsorption rate A correction term
*ω*	Water content (kg/kg)
*ρ_w_*	Density of water (kg/m^3^)
*m_w_*	The mass of water (kg)
*V_t_*	Total volume of soil sample (m^3^)
*V_w_*	Volume of total water in soil (m^3^)
$\rho_{d}^{s}$	Dry density of smectite (kg/m^3^)
*d_TOT_*	Thickness of TOT layer (9.5 Å)
*d_t_*	Basal spacing (Å)
*h_w_*	Total interlayer water thickness of soil
*V_imw_*	Micro pore volume/volume of interlayer water
*V_e_*	Macro pore volume/effect pore volume
*V_s_*	Volume of solid (m^3^)
*d_iw_*	Interlayer water thickness between two TOT layers
*Φ_macro_*	Macro porosity (Effective porosity)
*Φ_micro_*	Micro porosity (Interlayer porosity)
*ρ_s_*	Density of the solid (kg/m^3^)
*ρ_i_*	Density of the non-smectite minerals or impurities (kg/m^3^)
*G_s_*	Specific gravity of clay
*X_sm_*	The mass fraction of smectite in the bentonite
*L_e_*	Effective path length of flow
*L*	Sample length
*ref*	The reference case in which measurements are available
*β*	A fitting factor between Φ and Ψ in Pham et al. [92]
*σ*	Coefficient related to porosity and size of pore
*X_hs_*	Mole fraction of hydrated smectite
*M_sm_*	Molar mass of dry smectite (Kg/mol)
*ζ_c_*	The number of moles of waters in the interlayer adsorption or desorption reaction
*υ_il_*	The molar volume of the interlayer water (m^3^/mol)
*a, m*	Curve-fitting parameters by van Genuchten [24]
*n_max_, n_min_*	The maximum and minimum number of TOT layers of bentonite particle respectively
